# Ethnic considerations in the upper lip bite test: the reliability and validity of the upper lip bite test in predicting difficult laryngoscopy in Koreans

**DOI:** 10.1186/s12871-018-0675-5

**Published:** 2019-01-10

**Authors:** Jong Chan Kim, Yumin Ki, Jihee Kim, So Woon Ahn

**Affiliations:** Department of Anesthesiology and Pain Medicine, CHA Bundang Medical Center, CHA University, 59 Yatap-ro, Bundang-gu, Seongnam-si, Gyeonggi-do 13496 South Korea

## Abstract

**Background:**

Several methods have been used to predict difficult tracheal intubation. Among recently suggested methods, the upper lip bite test (ULBT) could serve as a good predictor. Soft tissue and skeletal hard tissue profiles are affected by many factors including ethnicity. We aimed to assess the clinical utility of the ULBT in Koreans while considering ethnic differences.

**Methods:**

Three-hundred-forty-four Korean patients undergoing general anesthesia with orotracheal intubation were included. Preoperatively, we recorded the patient’s Modified Mallampati (MMT) classification, ULBT ratings, and the Cormack–Lehane grade.

**Results:**

The area under the receiver operating characteristic (ROC) curve (AUC) was lower for the ULBT than the MMT (95% confidence interval: 0.0697–0.191, *p* < 0.0001). The ULBT showed high accuracy (73.83%) and specificity (98.04%). On the other hand, the ULBT showed significantly lower sensitivity (4.49%). Only nine of 344 Korean patients could not bite their upper lip; among them, only three presented a difficult laryngoscopic view.

**Conclusions:**

One factor related to the low sensitivity is the low incidence of a grade III ULBT in Koreans. In Asians, the scarcity of a grade III ULBT is explainable as a result of anteriorly displaced temporomandibular joints and redundant lip soft tissues. Despite its high specificity, the low sensitivity and AUC of the ULBT mean that the test results should be interpreted cautiously in Koreans. Ethnic differences should be considered when evaluating parameters related to soft tissues such as the ULBT.

**Trial registration:**

ClinicalTrials.gov Identifier: NCT01908218, Date of registration JUL 2013.

## Background

Predicting difficult laryngoscopic endotracheal intubation (TI) is an important concern for anesthesiologists. Anticipated difficulties offer opportunities to prepare alternative methods and use proper advanced management techniques. The Mallampati classification and other methods have been used to evaluate anatomical structures during the preoperative period for airway assessment.

Khan et al. suggested the upper lip bite test (ULBT) to, evaluates the ability of a patient to cover the mucosa of the upper lip with the lower incisors [1]. This simple bedside test was shown to have a good predictive value, specificity, and accuracy without the need for a light or sitting position [1, 2].

This method was based on cephalometric measurements, which differed in skeletal hard tissue and soft tissue profiles of Asian and Caucasian populations [3–10]. The ULBT evaluates mandibular movement, which reflects not only differences in skeletal hard tissue but also the conjointed movements of the ligaments, connective tissues, and soft tissues. In our experiences and other studies, there are some differences in ULBT in Koreans [11]. We suppose these discrepancies might be derived from cephalometric differences in Asian.

This study aimed to assess the differences in the ULBT in Koreans while considering ethnic differences. We tried to figure out the influence of the cephalometric differences to ULBT in Koreans.

## Methods

After obtaining approval from the Severance Hospital Institutional Review Board (IRB number: 4–2008-0583), the trial was performed at Yonsei University Severance Hospital.

The written informed consent was obtained from all subjects participating in the trial.

Three hundred forty-four Korean adult patients undergoing general anesthesia with orotracheal intubation were included in our registered prospective observational study. (ClinicalTrials.gov identifier: NCT01908218, Principal investigator: So Woon Ahn, Date of registration JUL 2013). This manuscript adheres to the applicable EQUATOR Network guidelines. Patients were excluded if they had facial anomalies, had temporomandibular(TM) joint disorder, were edentulous or required a rapid sequence induction. In the pre-anesthetic care unit, we recorded each patient’s age, sex, weight, height, American Society of Anesthesiologists (ASA) classification, Modified Mallampati (MMT) class [12], ULBT ratings [1], inter-incisor distance (With the mouth open maximally, measure the distance between the incisors, IID), thyromental distance (distance from the thyroid notch to the tip of the jaw, TMD), sternomental distance (distance from the chin (mentum) to the top of the notch of the thyroid cartilage, SMD) in sitting and fully head-extended position. The choice of the anesthesia induction technique was left to the attending anesthesiologist. After the loss of a response to a train- of -four or single- twitch ulnar nerve stimulation, laryngoscopy was performed by three skilled anesthesiologists (trained for at least four years, > 1000 endotracheal intubations) using a Macintosh laryngoscope with a size 3 or 4 blades. After obtaining a view of the glottis by direct laryngoscopy, the anesthesiologist assessed the Cormack - Lehane grade [13]. Grades 1 and two were considered as easy laryngoscopies. Grades 3 and 4 were considered difficult laryngoscopies from the inability to visualize the vocal cords. Grading was checked with no external laryngeal pressure. After the first attempted laryngoscopic view with Cormack –Lehane grade 3 or 4, external laryngeal pressure (backward upward rightward pressure maneuver [BURP]) was applied. [12] In the case of a second failed endotracheal intubation, an attempt with another intubation method such as fiber-optic bronchoscopy or video laryngoscopy assisted intubation was attempted and recorded.

Based on an institutional pilot study, the area under the receiver operating characteristic (ROC) curve (AUC)s of the MMT and ULBT were 0.61 and 0.52, respectively. We determined that 344 patients would be required to demonstrate a difference between two predicting tools with a type 1 error (α) of 5% and power (1-β) of 90%(two-sided) using the PASS program (NCSS, Kaysville, UT, USA).

For continuous variables, a Student’s t-test was used to assess differences in means between the groups. For categorical data, a chi-square test was used to assess differences in proportions across the categories. The cut-off points of thyromental, sternomental and mouth opening distances were obtained from the analysis on the ROC which was calculated maximize the sensitivity and specificity [14]. Each test, individually and together with various combinations, was evaluated by its calculated sensitivity, specificity, positive predictive value (PPV) and negative predictive value (NPV). A comparison of the predictability for each test was performed using AUCs. The significance of the difference between the two areas was assessed using the method described by Delong [15]. Statistical analysis was done by SPSS, version 22.0 (SPSS, Inc., Chicago, IL, USA) and Medcalc 14.8.1 (MedCalc Software, Ostend, Belgium).

## Results

**Fig. 1:**
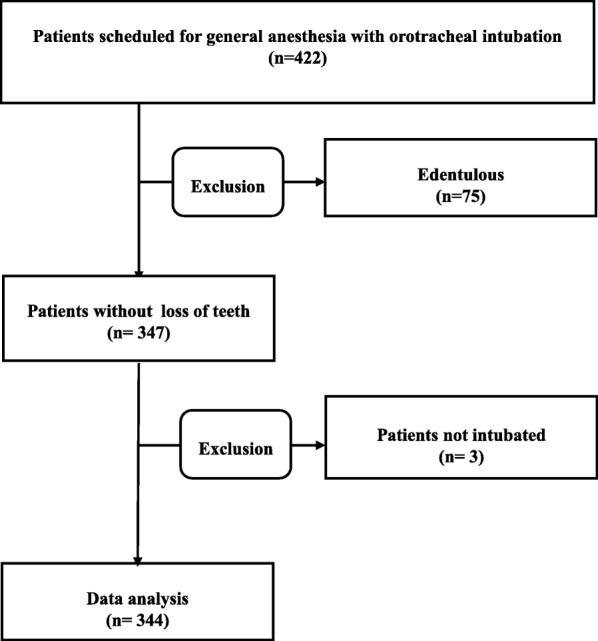
Flow chart of patient participation

Data were collected from four hundred forty-four elective surgical patients. Seventy-five patients were excluded because they were edentulous or the patients did not undergo TI because of changes in their anesthetic plans. A total of 344 patients’ data were analyzed (Fig. 1, Table 1).

**Table 1:** Patients’ characteristics

All patients *n* = 344	CL grade 1 & 2 = easy laryngoscopy *n* = 255	CL 3 & 4 = difficult laryngoscopy *n* = 89	*P* -value
Men [n (%)]	104 (68%)	49 (32%)	0.025‡
Women [n (%)]	151 (79.1%)	40 (20.9%)	
Age (yr)	43.17 ± 15.26	51.13 ± 15.19^*^	< 0.001
Height (cm)	162.86 ± 9.91	163.45 ± 8.99	0.786
Weight (Kg)	62.80 ± 11.60	62.70 ± 9.42	0.764
Body mass index (kg/m^2^)	23.60 ± 3.32	23.45 ± 2.75	0.784^†^
ASA			
1	152(59.6%)	31(34.8%)	
2	97(38.0%)	56(62.9%)	
3	6(2.4%)	2(2.2%)	
Inter-Incisor distance (mm)	45.45 ± 7.20	41.15 ± 6.07^*^	<.0001^†^
Thyromental distance (mm)	84.18 ± 15.30	78.98 ± 11.26^*^	0.0002^†^
Sternomental distance (mm)	182.33 ± 22.38	173.83 ± 19.38^*^	0.003^†^

Values are number (proportion) or mean ± standard deviation; ‡, The Chi-square test; §, The Fisher exact test; †, The Mann–Whitney U test.CL grade: Cormack-Lehane grade

During direct laryngoscopy, 89 patients presented with a difficult laryngoscopic view (Cormack–Lehane grade of 3 or 4) without external manipulation (Table 2). Among these patients, 86 could be intubated by applying external laryngeal pressure, and two required alternative techniques. One patient was intubated using video laryngoscopy (Glidescope®, Saturn Biomedical Systems, Burnaby, BC, Canada) and another patient was intubated with the use of fiber-optic devices. Moreover, one patient was intubated successfully after multiple laryngoscopic trials while waiting for the preparation of alternative devices.

**Table 2:** Relationship between pre-anaesthetic assessment classifications and Cormack and Lehane grade

	Cormack and Lehane grade		
Pre-anaesthetic test	1 & 2(%) (n = 255)	3 & 4(%) (n = 89)	*p*
Modified Mallampati			
Class 1 & 2	156(61.2)	29(32.6)	<.001
Class 3 & 4	99(38.8)	60(67.4)	
Upper lip bite			
Class 1 & 2	250(98.0)	85(95.5)	0.245
Class 3	5(2.0)	4(4.5)	

Values are number (proportion); ^*^ The Chi-square test

An MMT class > II, IID ≤ 4.5 cm, TMD ≤ 8.3 cm, and SMD ≤ 17.9 cm were defined as cut-off points for difficult intubation (Table 3).

**Table 3:** Receiver operating characteristic (ROC) analysis for difficult intubation

Variables	AUC Value (95% CI)
IID ≤ 4.5 cm	0.664 (0.611–0.713)
TMD ≤ 8.3 cm	0.622 (0.568–0.673)
SMD ≤ 17.9 cm	0.629 (0.576–0.680)
MMT	0.643 (0.590–0.694)
ULBT	0.513 (0.458–0.567)

Values are number (confidence interval). *IID* inter-incisor distance, *TMD* thyromental distance, *SMD* Sternomental distance (SMD), *MMT* Modified Mallampati test, *ULBT* upper lip bite test, *AUC* areas under the ROC curves, *CI* confidence interval

Table 4 shows the true positive, false positive, true negative, false negative, accuracy, sensitivity, specificity, PPV, and NPV; moreover, AUCs obtained from the ROC analysis are shown for the MMT and ULBT (Appendix).

**Table 4:** Predictive values for the Upper lip bite test (ULBT) and the modified Mallampati test (MMT) to predict the occurrence of a grade 3 or 4 according to the Cormack-Lehane grade

Outcome of calculations	ULBT (*n* = 344)	MMT (*n* = 344)
True-Positive	4	60
False-Positive	5	99
True-Negative	250 (74.63%)	156 (84.32%)
False-Negative	85 (25.37%)	29 (15.68%)
Accuracy (%)	73.83	62.80
Sensitivity (%)	4.49	67.42
Specificity (%)	98.04	61.18
Positive prediction value (%)	44.44	37.74
Negative Prediction value (%)	74.63	84.32
AUC of ROC-curve	0.513	0.643

Values are number (proportion)

**Fig. 2:**
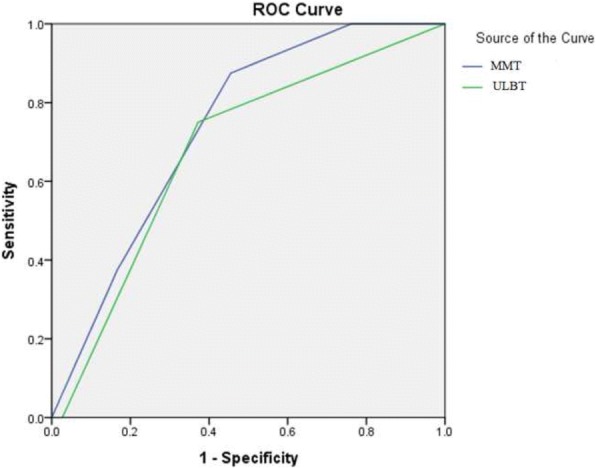
ROC curves of MMT and ULBT

The AUC, the primary endpoint of this trial, was lower for the ULBT than the MMT (the difference between the areas: 0.13, 95% confidence interval: 0.0697–0.191, *p* < 0.0001, Table 3, Fig. 2).

In our study, the accuracy of the ULBT (73.83%) was higher than that of the MMT (62.80%), and the specificity of the ULBT (98.04%) was higher than that of the MMT (61.18%). Particularly, the ULBT showed significantly lower sensitivity (4.49%) compared with that of the MMT in our trial (67.42%). The prevalence of a difficult laryngoscopy (DL) was 25.87% (89 of 344), and the percentage of patients with an MMT class > II was 67.4% (60 of 344), while only nine patients (2.6%) showed a grade III ULBT (Table 2).

## Discussion

Our study demonstrates the ULBT shows particularly high specificity, low false-positive rates and high accuracy in Koreans. The differences were due to lower incidence of high-grade ULBT in Korean than in other ethnities [2, 16, 17]. According to a review of several works of literature, such character might be explainable with the soft tissue redundancy and skeletal variance in Far East Asians.

Several methods such as the MMT classification, IID, TMD, and SMD have focused on one or more patient-related factors that may identify those at risk for a difficult TI before the induction of anesthesia [16, 18–20]. Concerning applicability, the ULBT does not require an additional light, restriction of phonation, or for the patient to be in the sitting position. The ULBT has been used as a simple bedside test for a DL with good predictive accuracy. It could serve as a good predictor for difficult laryngoscopic intubation because the range and freedom of mandibular movement and the architecture of the teeth have pivotal roles in facilitating laryngoscopic intubation [1]. The ULBT showed higher accuracy (75.9–91%) in previous studies than the MMT (63.7–67.7%) [1, 2, 21, 22] and good predictability with a high AUC value (0.604–0.85) [1, 2, 22]. The percentage of correctly predicted easy laryngoscopies among all laryngoscopies (the specificity) of the ULBT was very high (82.35–92.5%) [1, 2, 23, 24].

The nature of the soft tissue profile is affected by many factors other than the skeletal hard tissue profile, including ethnicity. The ULBT evaluates the range and freedom of mandibular movement and the architecture of the teeth. Mandibular movement is the conjoined movements of skeletal hard tissue, ligament, and soft tissue [25]. Furthermore, the ULBT classification is based on the upper lip mucosa, which is soft tissue [1].

From our observational results comparing the ULBT with the MMT, which has been used widely, the AUC of the MMT in Koreans was 0.627, which is indicative of unreliable predictability similar to those found in recent meta-analyses [5, 26]. However, the AUC of the ULBT was 0.519, which implies predictability that is much lower than those in other studies of different ethnicities (0.604–0.826) [1, 2, 27]. The NPV of the MMT is higher than that of the ULBT. A high AUC value and high NPV mean that the MMT is highly reliable and likely to detect an easy laryngoscopic view. The ULBT has a lower PPV than the MMT. Therefore, many positive results from this procedure are false positives. However, the PPV and NPV are not intrinsic and also depend on the prevalence, which was very low for positive results. In this trial, the prevalence of high-grade ULBT values was 2.6% (9 of 344). This result is also similar to that of another study on Koreans (16 of 305) [11]. We speculated that differences in the soft tissue and bony structure in line with ethnic differences might explain the differences between the ULBT and MMT results. Our results also indicated that the ULBT has higher accuracy and specificity than the MMT. However, sensitivity was much lower (4.49%) compared with that of the MMT in our trial (67%) and consistent with ULBT data from previous trials (28.2–76.5%) [1, 2, 21, 22, 28]. This means that many patients with a DL will not be identified by the ULBT (a large number of patients will have false-negative tests).

$$ \mathrm{Sensitivity}=\frac{\mathrm{Number}\kern0.17em \mathrm{of}\ \mathrm{true}\ \mathrm{positives}}{\mathrm{Number}\ \mathrm{of}\ \mathrm{true}\ \mathrm{positives}+\mathrm{Number}\ \mathrm{of}\ \mathrm{false}\ \mathrm{negatives}} $$

We deduced that one of the factors causing the low sensitivity was the low incidence of a grade III ULBT in Korean subjects. In our study, only nine patients of 344 could not bite their upper lip, and only three of them presented a difficult laryngoscopic view. In Korea, a grade III ULBT is rarely observed [11]. There can be several reasons for this, with ethnic cephalometric differences possibly being one reason. Several studies were published comparing soft tissues between different ethnic groups in orthodontics and the field of maxillofacial surgery [3, 4, 6, 7, 10, 29–32].

In a comparison of Southern Chinese and British Caucasian cephalometric standards, Chinese upper lips were longer with a more acute angulation than those of Caucasians [7]. Moreover, Korean subjects had a lower angle of nasal inclination, and a higher degree of lip protrusion compared with European-American adults and the upper and lower lips were positioned more anteriorly [3]. Chang and colleagues conducted a morphometric analysis comparing Asian and European-American subjects [32]. Far East Asian (Chinese, Japanese, Korean, and Taiwanese) men seemed to have a significantly shorter cranium and smaller anterior cranial base angles. Compared with Caucasians, Asians with clinically acceptable occlusions tended to have a shorter midface, prominent mandibles, and an anteriorly displaced TMJ in the posterior cranial base [32]. It was also noted that these features resulted from the relative retrusion of the nasomaxillary complex and the relatively forward position of the mandible.

Therefore, in Asians, the scarcity of a grade III ULBT is explainable as a result of an anteriorly displaced TMJ and redundant soft lip tissues. It is clear that the ULBT shows a much lower false-positive rate, lower sensitivity, and higher PPV than other predictive methods.

## Limitations

One of the limitations of a test to predict a DL is the discrepancy between a DL and difficult intubation. Patients who presented with a DL (Cormack–Lehane grade 3 or 4) could easily be classified as a grade II or better with the application of external pressure to the larynx (the BURP maneuver) to move the epiglottis. These patients would have been described as having an easy TI. Therefore, it is difficult to predict a difficult TI (DTI) only by mandibular movement, and a DL does not predict a DTI.

Moreover, in this trial, the prevalence of a DL was 25.87% (89 of 344), and the DTI/DL ratio was 3.4% (3/89). These are similar to the results shown in previous studies (4.3–77.8%) [20, 33, 34]. And, even though we used nerve stimulation for checking the achievement of muscle relaxation, the methodological limitation might exist cause we didn’t protocolize the technique of anesthetic induction, so it is possible that direct laryngoscopy and endotracheal intubation has not been performed under the same conditions in all patients. We didn’t collect the data of surgery that patients underwent. It could be comparable, but we just wanted to evaluate the predictability of ULBT and had excluded patients who had considerable abnormalities to airway prediction and evaluation.

## Conclusions

In Koreans, the ULBT shows very high specificity. The ULBT presented low false-positive rates and high accuracy. Cephalometric ethnical differences may present in Far East Asians are one reason. Therefore, especially in Asians, soft tissue redundancy and skeletal variance should be considered based on ethnic differences when evaluating parameters related to soft tissue such as the ULBT.

## Abbreviations

ASA: American Society of Anesthesiologists
AUC: The area under the receiver operating characteristic curve
BURP: Backward upward rightward pressure maneuver
DL: Difficult laryngoscopy
DTI: Difficult tracheal intubation
IID: Inter-incisor distance
MMT: Modified Mallampati test
NPV: Negative predictive value
PPV: Positive predictive value
ROC: Receiver operating characteristic
SMD: Sternomental distance
TI: Tracheal Intubation
TMD: Thyromental distance
ULBT: The upper lip bite test

## References

[1] Khan ZH, Kashfi A, Ebrahimkhani E (2003). A comparison of the upper lip bite test (a simple new technique) with modified Mallampati classification in predicting difficulty in endotracheal intubation: a prospective blinded study. Anesth Analg.

[2] Eberhart LH, Arndt C, Cierpka T, Schwanekamp J, Wulf H, Putzke C (2005). The reliability and validity of the upper lip bite test compared with the Mallampati classification to predict difficult laryngoscopy: an external prospective evaluation. Anesth Analg.

[3] Hwang HS, Kim WS, McNamara JA (2002). Ethnic differences in the soft tissue profile of Korean and European-American adults with normal occlusions and well-balanced faces. Angle Orthod.

[4] Ioi H, Nakata S, Nakasima A, Counts AL (2007). Comparison of cephalometric norms between Japanese and Caucasian adults in antero-posterior and vertical dimension. Eur J Orthod.

[5] Lee A, Fan LT, Gin T, Karmakar MK, Ngan Kee WD (2006). A systematic review (meta-analysis) of the accuracy of the Mallampati tests to predict the difficult airway. Anesth Analg.

[6] Miyajima K, McNamara JA, Kimura T, Murata S, Iizuka T (1996). Craniofacial structure of Japanese and European-American adults with normal occlusions and well-balanced faces. Am J Orthod Dentofac Orthop.

[7] Cooke MS, Wei SH (1989). A comparative study of southern Chinese and British Caucasian cephalometric standards. Angle Orthod.

[8] Park IC, Bowman D, Klapper L (1989). A cephalometric study of Korean adults. Am J Orthod Dentofac Orthop.

[9] Wu J, Hagg U, Rabie AB (2007). Chinese norms of McNamara's cephalometric analysis. Angle Orthod.

[10] Alcalde RE, Jinno T, Pogrel MA, Matsumura T (1998). Cephalometric norms in Japanese adults. J Oral Maxillofac Surg.

[11] Seo SH, Lee JG, Yu SB, Kim DS, Ryu SJ, Kim KH (2012). Predictors of difficult intubation defined by the intubation difficulty scale (IDS): predictive value of 7 airway assessment factors. Korean J Anesthesiol.

[12] Samsoon GL, Young JR (1987). Difficult tracheal intubation: a retrospective study. Anaesthesia.

[13] Cormack RS, Lehane J (1984). Difficult tracheal intubation in obstetrics. Anaesthesia.

[14] Perkins NJ, Schisterman EF (2006). The inconsistency of "optimal" cutpoints obtained using two criteria based on the receiver operating characteristic curve. Am J Epidemiol.

[15] DeLong ER, DeLong DM, Clarke-Pearson DL (1988). Comparing the areas under two or more correlated receiver operating characteristic curves: a nonparametric approach. Biometrics.

[16] Mahmoodpoor A, Soleimanpour H, Golzari SE, Nejabatian A, Pourlak T, Amani M, Hajmohammadi S, Hosseinzadeh H, Esfanjani RM (2017). Determination of the diagnostic value of the modified Mallampati score, upper lip bite test and facial angle in predicting difficult intubation: a prospective descriptive study. J Clin Anesth.

[17] Myneni N, O'Leary AM, Sandison M, Roberts K (2010). Evaluation of the upper lip bite test in predicting difficult laryngoscopy. J Clin Anesth.

[18] Mallampati SR, Gatt SP, Gugino LD, Desai SP, Waraksa B, Freiberger D, Liu PL (1985). A clinical sign to predict difficult tracheal intubation: a prospective study. Can Anaesth Soc J.

[19] Tse JC, Rimm EB, Hussain A (1995). Predicting difficult endotracheal intubation in surgical patients scheduled for general anesthesia: a prospective blind study. Anesth Analg.

[20] Butler PJ, Dhara SS (1992). Prediction of difficult laryngoscopy: an assessment of the thyromental distance and Mallampati predictive tests. Anaesth Intensive Care.

[21] Hester CE, Dietrich SA, White SW, Secrest JA, Lindgren KR, Smith T (2007). A comparison of preoperative airway assessment techniques: the modified Mallampati and the upper lip bite test. AANA J.

[22] Khan ZH, Mohammadi M, Rasouli MR, Farrokhnia F, Khan RH (2009). The diagnostic value of the upper lip bite test combined with sternomental distance, thyromental distance, and interincisor distance for prediction of easy laryngoscopy and intubation: a prospective study. Anesth Analg.

[23] Salimi A, Farzanegan B, Rastegarpour A, Kolahi AA (2008). Comparison of the upper lip bite test with measurement of thyromental distance for prediction of difficult intubations. Acta Anaesthesiol Taiwanica.

[24] Faramarzi E, Soleimanpour H, Khan ZH, Mahmoodpoor A, Sanaie S (2018). Upper lip bite test for prediction of difficult airway: a systematic review. Pak J Med Sci.

[25] Sahin SH, Yilmaz A, Gunday I, Kargi M, Sut N, Taskinalp O, Ulucam E (2011). Using temporomandibular joint mobility to predict difficult tracheal intubation. J Anesth.

[26] Lundstrom LH, Vester-Andersen M, Moller AM, Charuluxananan S, L'Hermite J, Wetterslev J (2011). Poor prognostic value of the modified Mallampati score: a meta-analysis involving 177 088 patients. Br J Anaesth.

[27] Huh J, Shin HY, Kim SH, Yoon TK, Kim DK (2009). Diagnostic predictor of difficult laryngoscopy: the hyomental distance ratio. Anesth Analg.

[28] Safavi M, Honarmand A, Zare N (2011). A comparison of the ratio of patient's height to thyromental distance with the modified Mallampati and the upper lip bite test in predicting difficult laryngoscopy. Saudi J Anaesth.

[29] Liu Y, Lowe AA, Zeng X, Fu M, Fleetham JA (2000). Cephalometric comparisons between Chinese and Caucasian patients with obstructive sleep apnea. Am J Orthod Dentofac Orthop.

[30] Porter JP, Lee JI (2002). Facial analysis: maintaining ethnic balance. Facial Plast Surg Clin North Am.

[31] Solmaz I, Raberin M (2011). Is the ethnic factor an orthodontic therapeutic instructor?. Orthod Fr.

[32] Chang HP, Liu PH, Tseng YC, Yang YH, Pan CY, Chou ST (2014). Morphometric analysis of the cranial base in Asians. Odontology.

[33] Frerk CM (1991). Predicting difficult intubation. Anaesthesia.

[34] Wajekar AS, Chellam S, Toal PV (2015). Prediction of ease of laryngoscopy and intubation-role of upper lip bite test, modified mallampati classification, and thyromental distance in various combination. J Family Med Prim Care.

